# The influenza A(H1N1) epidemic in Mexico. Lessons learned

**DOI:** 10.1186/1478-4505-7-21

**Published:** 2009-09-28

**Authors:** José A Córdova-Villalobos, Elsa Sarti, Jacqueline Arzoz-Padrés, Gabriel Manuell-Lee, Josefina Romero Méndez, Pablo Kuri-Morales

**Affiliations:** 1 Mexican Ministry of Health, Mexico; 2 Mexican Society of Public Health, Mexico; 3 School of Medicine, National Autonomous University, Mexico City, Mexico

## Abstract

Several influenza pandemics have taken place throughout history and it was assumed that the pandemic would emerge from a new human virus resulting from the adaptation of an avian virus strain. Mexico, since 2003 had developed a National Preparedness and Response Plan for an Influenza Pandemic focused in risk communication, health promotion, healthcare, epidemiological surveillance, strategic stockpile, research and development. This plan was challenged on April 2009, when a new influenza A(H1N1) strain of swine origen was detected in Mexico. The situation faced, the decisions and actions taken, allowed to control the first epidemic wave in the country. This document describes the critical moments faced and explicitly point out the lessons learned focused on the decided support by the government, the National Pandemic Influenza Plan, the coordination among all the government levels, the presence and solidarity of international organizations with timely and daily information, diagnosis and the positive effect on the population following the preventive hygienic measures recommended by the health authorities. The international community will be able to use the Mexican experience in the interest of global health.

## Introduction

Health threats have occurred throughout the history of the populations in the world. The epidemiologic, demographic and risk transition processes determine changes in the morbidity and mortality profiles of the populations. Moreover, both the globalization and the environmental impact involving climatic repercussions have accelerated these changes. Throughout the history of mankind, great pandemics have been documented; suffice to remember those caused by the Black Death, small pox, cholera and influenza. The latter, described as early as the Hippocratic times, caused three large pandemics during the 20^th ^century: the one in 1918-19, known as the "Spanish flu", the one in 1958-59, and finally the one in 1968 [1,2].

In particular, the 1918-19 pandemic caused, according to estimates, between 40 and 100 million deaths at a time when the communications and means of transportation on earth were not as fast and efficient as they are now.

The magnitude of the public health impact at the global level, the associated social and economic consequences, the observed trends and the periodicity of the influenza pandemics led the World Health Organization to support and issue the recommendation among its member states of developing their respective preparedness plans for this threat [3,4].

In Mexico the interinstitutional work plan to develop the National Pandemic Influenza Preparedness and Response Plan was established in 2003. The plan considers, among others, the Multisectoral Operating Strategy, which represents its translation into the specific actions to be developed by each institution before the probable emergency of a new pandemic [5,6]. On the other hand, in view of the persistent circulation of the seasonal influenza virus, Mexico was one of the first countries to include, as of 2004, this biologic in its immunization regimen, focusing on the high risk groups, mainly children and people over 60 years of age.

The Preparedness and Response Plan was structured around various scenarios stemming from the events of 1918-19, based on which the federal reserve of medications (more than 1,000,000 treatment courses of oseltamivir) and supplies was created, the healthcare guidelines were prepared, together with those for epidemiologic surveillance and diagnosis, and the messages to be conveyed to the population to contain the influenza pandemic [3,4].

Within this setting, and as per the available information, it was assumed that the pandemic would emerge from a new human virus resulting from the adaptation of an avian virus and that it would very likely originate in the Asian continent, given that the latter was already being affected and continued to have cases of influenza virus A(51N1); more than 450 cases were documented among humans with a fatality rate of more than 60% [5]. However, it was also thought that the new pandemic could originate from other strains and that it could start anywhere in the world, as was the case of the new A(H1N1) strain of a swine, avian and human origin. Thus in April 2009 the socio-epidemiologic and biologic reality tried us [6].

The situation faced by Mexico and the decisions made are a topic of analysis and allow us to better prepare ourselves for a new public health threat. This document intends to succinctly describe the critical moments faced and explicitly point out the lessons learned. The international community will be able to use the Mexican experience in the interest of global health.

## The epidemiologic alert

In early April an atypical situation in the behavior of acute respiratory tract infections was seen in Mexico, characterized by an increased duration of transmission of seasonal influenza, as well as by an enlarged number of admissions during the spring, mainly in Mexico City, its conurbation with the State of Mexico, and the State of San Luis Potosí and Oaxaca. Likewise, the fact that young adults were the affected age group, together with the isolation of an influenza A virus that could not by typed in the reference laboratory, was striking [6].

Due to the former, and according to the National Pandemic Influenza Preparedness and Response Plan, an epidemiologic alert was issued in the entire country on April 17, 2009. The alert recommended all the federate entities to intensify their epidemiologic surveillance actions aimed at detecting cases suspicious of unusual severe influenza or pneumonia, with the corresponding pharyngeal smears. At the same time, the communication channels were reinforced to increase the collaboration with international institutions.

On April 13, 2009 in the capital city of Oaxaca, a southern state, a 39-year-old female died of severe atypical pneumonia. A local laboratory diagnosed a coronavirus as a causative agent, leading health authorities to send the sample to the United States Centers for Disease Prevention and Control (CDC). Later, when additional cases of atypical pneumonia were reported, samples were shipped to the Winnipeg laboratory in Canada.

The influenza continued to behave atypically and, 5 days after the epidemiologic alert, the necessary steps were taken to prevent the population from attending crowded places, and the use of the "etiquette sneeze" and frequent hand washing were recommended as preventive measures. On April 23, the laboratory tests fully identified the virus as influenza A(H1N1) from a virus strain unknown until then, which meant that its behavior, virulence, transmission capacity and origin were all unknown. And initially, even its susceptibility to the available antivirals, the magnitude of the associated risk and its pandemic potential were also unknown. The previous experience of other influenza pandemics in other countries was the only source of knowledge.

It was also thought that as difficult as it was to stop the spread of the virus, there was a good chance of slowing down transmission rate and mitigating the consequences. Therefore, that evening a "state of sanitary contingency" was declared and informed to the population, a prepandemic alert was declared, and the preventive and control measures were intensified. This meant cancelling all education activities in the Federal District and its entire metropolitan area, and in the State of Mexico; the State of San Luis Potosí decided to implement these same measures. Three days later, social distancing measures were implemented in the rest of the country, particularly the suspension of school-based activities.

On April 24, the President of the Republic issued a decree empowering the Federal Minister of Health to coordinate the public, private and social settings to comply with the national ordinance concerning various general health actions aimed at preventing, controlling and fighting the existence and transmission of the recently detected influenza H1N1 virus. The next step was summoning an Extraordinary Meeting of the General Health Council, as well as the National Health Council, to establish and coordinate all the prevention and health promotion actions, and those aimed at containing the epidemic and providing healthcare. During the following days other measures were implemented consisting of the suspension of all sorts of events held indoors or outdoors, whether at religious centers, stadiums, theaters, cinemas, bars, discotheques, that gatehered large groups of people as well as all the activities of the federal public administration, except for those that, according to the agencies themselves, were necessary to assure an appropriate, timely and continuous service provision. The suggestion was also made to interrupt the unessential services of the productive sectors and to maintain only those necessary for families to have basic supplies available, like food, water, electricity and transportation, among others.

As the epidemic evolved, its behavior and effects were analyzed and decisions aimed at restoring the country's economic, social and educational life were made. Thus 13 days after the onset of the sanitary contingency, the public, private and social work life went gradually back to normal, and the mid-higher and higher education academic institutions resumed their activities. Four days later the primary education activities were gradually regularized in most of the country, according to the specific circumstances in each federate entity, particularly considering the number of new cases.

## The National Response

Once the sanitary emergency was declared, various coordination mechanisms were established among all the areas involved in healthcare to contain in a timely and organized way this emergency and, at the same time, reduce as much as possible the negative impact of the influenza H1N1 epidemic on the health of the Mexican population.

To this end, regular meetings were scheduled with the participation of the federal, state and municipal government levels, and the secretariats of state. As per the legal framework applicable in view of this sanitary contingency, the Ministry of Health convened an extraordinary and permanent meeting of the General Health Council and the National Health Council. The former reports to the President of the Republic and has regulatory and advisory roles; the latter is a collegiate body responsible for formulating the health policies implemented in the Mexican Republic and integrates horizontally and democratically the country's 32 federate entities, it is presided by the Minister of Health and the sanitary heads of public, private, academic and social sectors, thereby the decision was made to include in the Response Plan the following six broad dimensions for facing the epidemic:

### Risk communication

An effective communication plan was established targeted to the general population, healthcare workers and the information media. For this purpose press conferences were held on a daily and ongoing basis, with the support of all the mass media, including the internet and telephone lines. The press conferences were presided by the country's Minister of Health and by the health authorities in each of the federate entities, who informed the population, in real time, about the status of the epidemic in a practical, ongoing and effective manner. Additionally, information was distributed to the academia and the public and private institutions in the country.

To address questions from the general public and provide them guidance on the healthcare and psychological support services, a toll free telephone number was made available 24 hours a day. More than 5 million calls with questions were received.

After the first few days, the civil population would wait for the press conferences delivered by the Minister of Health with national coverage. This led to unification of the knowledge and the statistical data, contributed to answer the questions of the society, helped the people remain calm at that time of crisis and, mostly, gained the support of the population in complying with the epidemic-related recommendations [7].

### Health promotion

This activity was intended to affect the positive and contain the negative health determinants, contributing to a better control of people over their health. Through the mass media and the distribution of brochures, posters and fliers emphasis was made on the use of masks, frequent hand washing, the use of alcohol gel, the "etiquette sneeze", the use of disposable tissues and their proper and hygienic disposal, avoiding overcrowded and/or closed places, and not leaving the home, unless it was necessary [8]. It is worthwhile mentioning that the civil population acted responsibly and was solidary with the health authorities in view of the possibility of a major catastrophe. The population of one of the largest cities in the world adopted the suggested steps even though they touched the most sensitive fibers of the social fabric and affected the production of goods and services.

### Healthcare

Initially the aim was to assure the protection of the personnel participating in the teams that provided medical care. Measures were taken to guarantee the supply and availability of the supplies they required to perform their activities [9]. All the public healthcare institutions throughout the country opened their doors so that anyone considered as a suspicious case of influenza, could request healthcare that included the diagnosis and further treatment with the antiviral agent oseltamivir. The latter proved to work properly and to modify the patients' clinical picture, particularly when administered within 72 hours of the onset of symptoms.

Technical and procedures manuals were distributed to the healthcare services containing the working definitions of suspicious case, probable case and confirmed case, together with the necessary protocols for care, collection of biological samples, treatment and reporting of cases, thus implementing a triage system for the appropriate classification of patients [7,10].

Contacts of all the confirmed cases of influenza virus A(H1N1) were visited and offered prophylaxis with oseltamivir; an intensive case search was conducted by trained personnel that traveled in healthcare mobile units (health caravans) and who also delivered informative talks, did quick diagnostic tests and participated in the health promotion activities conducted in strategic zones [10,11].

At the beginning of the crisis a biological sample was collected from all suspected cases of influenza for confirmation purposes. However, given that the influenza virus A(H1N1) was confirmed in 30% of the samples, the sample collection flow charts were modified trying to be more selective. Moreover, a "quick test" was purchased and distributed massively during the crisis; it helped meet the need for an immediate diagnostic support, for purposes of deciding on treatment administration.

### Epidemiologic Surveillance

This activity focused on two large areas. The first, and most important one, consisted of raising the awareness of the population by means of information dissemination through the mass media so that, if anyone of any age had the cardinal symptoms, i.e., fever, cough or respiratory distress (suspicious case), they would go to the institutional healthcare services in the country.

The second one consisted of collecting information on the evolution of the epidemic. Two epidemiologic surveillance systems were set up to get basic descriptive information of the cases, including the time, the place, and the individual. The purpose of the first system was recording the suspected cases of influenza by collecting the above mentioned data, as well as additional information about the patients, like their health status, medical and healthcare history, whether they were hospitalized or not, date of onset and resolution of the disease, probable diagnosis, treatment provided, information of their contacts, and so on. The National Epidemiological Surveillance System (SINAVE) obtained information "on line" using access codes and passwords and represented the basis of the statistical information. The second system was established to ship biological samples to the reference lab. It led to having a nominal registry of probable influenza cases from whom a pharyngeal smear was taken (by definition, a probable case was considered as a suspicious case with a biological sample). The date of onset and the main symptoms as well as the sample date, besides the above mentioned time, place and individual data were obtained. This system, called Influenza Surveillance System (SISVFLU), allowed identifying the confirmed cases of influenza A(H1N1), the cases of influenza A, those caused by other agents, and the negative cases [12].

It is a fact that during the early days we did not have, as neither did almost any other country in the world, the capability of identifying this new pathogen because the essential "primer" to make the diagnosis was not available in the market. However we did have a broad network of laboratories certified by the World Health Organization (WHO), which made it possible to set up the appropriate equipment and technique for viral identification in only three days time. The pieces of equipment were strategically placed within the lab network to extend the regional coverage as necessary.

At the same time, a nominal registry was kept containing the patient records and death certificates of all the cases reported as compatible with influenza. Those records were thoroughly reviewed by an expert group and resulted in the registry of the deaths caused by the influenza virus A(H1N1).

Based on the information collected - initially of the probable and confirmed cases and the confirmed deaths, and then of the suspicious cases - it was possible to analyze the epidemic behavior on a daily basis and thus make the corresponding decisions with the proper rationale.

### Strategic Stockpile

As per the National Pandemic Influenza Preparedness and Response Plan [5], Mexico had personal protection equipment, guidelines for the clinical management of cases, educational and promotional materials, stores of medications (antibiotics) and antivirals (oseltamivir and zanavivir), as well as other supplies that were essential to providing timely and appropriate care. The medication stores were supplied to the different federate entities based on their needs. The available stockpile was reinforced with the supplies received from several countries during the critical phase, which were also distributed according to each state's specific needs. All the supplies and medications were distributed throughout the health sector institutions and to the population at no charge.

### Research and Development

Different groups of national and international researchers devoted themselves to studying the virus and characterizing it genetically and antigenically [13]. This information was provided to WHO for its most appropriate and convenient use, particularly for producing a vaccine, foreseeing needs to protect the poorest countries. The virus' phylogenetic tree was also determined [6]. Moreover, the epidemic behavior was characterized as well for the benefit of human beings and with the purpose of upgrading the measures to reduce the spread of the disease considering its pandemic course.

A fund was created to provide economic incentives to the academic and researcher groups to participate in grants to further the knowledge on the virus, its virulence, transmissibility, affected groups, severity, etc.

## Response after the critical phase

The analysis of the epidemiologic behavior led to concluding that Mexico had overcome the critical phase of the influenza A(H1N1) epidemic and therefore the government prepared itself to normalize the activities that had been disrupted by it. To this end, the prevention and control guidelines for resuming the activities at schools, work places, public transportation, and meeting centers were disseminated. This enabled to resume the activities of the public administration and the non-essential services provided by the productive and restaurant sectors and most meeting places [7]. The establishment of checkpoints at schools is a measure that led to maintaining a low case number and breaks the transmission chain. Their purpose is to timely detect the suspicious cases, refer them to the healthcare services for proper management, and start recording and examining their contacts.

All the players involved are aware that complete control has not been achieved in all the states, but the trend of the epidemic at the national level continues to be downward. The citizenship was therefore asked to keep guard implementing the preventive and health promotion actions. Moreover the recommendation was made to follow the basic hygiene measures of hand washing, "etiquette sneeze", going to the doctor in case of suspicious symptoms, avoiding, to the extent possible, hand shaking and kissing to greet people, using a face mask only in uncontrolled crowded places, like public transportation, and maintaining the school health checkpoints. The autonomy of the federate entities was further supported to allow them implementing the best strategy in case of outbreaks. A focused control was suggested, together with the temporary closing of the schools where new cases are detected.

Considering the major potential impact of this viral infection on health and human activities worldwide, and given that the most effective means to eventually control it is a vaccine, the latter was considered as a social and global asset. Therefore Mexico, through WHO, donated the strain of this new virus to the world, to prevent the existence of a patent and reduce costs, and to develop a vaccine to control and prevent this new type of human influenza, which could become a major catastrophe that would further complicate the possibilities of human development, mainly in the poorest and unprotected populations.

## The impact

As difficult as it may be to answer the question of what would have happened if the difficult decisions made had not been made, it is nevertheless possible to outline hypothetical scenarios.

There are various useful approaches to model the potential impact of a pandemic. All of them are based on assumptions stemming from the documentation of previous influenza pandemics, particularly in the 20^th ^century. Since the purpose of models is to anticipate unknown situations, the former should therefore be taken with a grain of salt. Moreover, they include only a few of the impact indicators of this kind of pandemia [6,14,15].

It is estimated that, in an extreme scenario, such as the one in 1918-19, in a period of 8 - 10 weeks, 50,000 additional deaths and more than 240,000 additional hospital admissions could have occurred, as well as an excess of 14 million medical consultations [16]. Fortunately, this scenario did not occur.

A moderate scenario, such as the 1968 pandemic, foresaw that without any mitigation and control measures, hospital admissions would have exceeded 30,000, deaths would have amounted to 8,600 and almost 4.6 million outpatient consultations would have occurred [9,16].

The fact is that up to August 28, 2009, only 187 deaths and no more than 1,000 hospital admissions have been reported [7]. We now know that most of the people who died were 20-49 years old (65%). The major symptoms were cough (86%), fever (85%), dyspnea (74%), expectoration and malaise (52% and 48%, respectively), followed by myalgia (27%), headache (25%) rhinorrhea and cyanosis (23% each), hemoptysis (19%), odynophagia (18%) chest pain (14%), and the following in less than 10%: vomiting, nasal obstruction, conjunctival hyperemia and diarrhea. Of the 187 patients who have so far died of influenza A(H1N1), 35% of them had a history of metabolic conditions like obesity and diabetes mellitus, 23% of smoking and 16% of cardiovascular disease, e.g., angina and arterial hypertension, followed by respiratory tract and infectious conditions. The reported fatality rate is 0.87%, the incubation period is 3-4 days, the transmission rate reported by Ro is 1.4 and the confirmation rate of suspicious cases is 29% [7].

These figures undoubtedly show that the measures taken had an important repercussion on population health, compared with the data of the above mentioned models. The actions taken contributed to contain both the magnitude and the rate of disease spread and allowed to save valuable time to understand the virulence and transmission features of the virus. This time helped other countries to better prepare themselves and helped the Mexican population to stop the transmission chains; this time also showed that in the presence of threats to the population integrity, most important of all is to protect the people's health since, regardless of the impact of this sanitary response on other realms of the national life, we are not counting deaths by the thousands and the spread of the virus within our country is under control.

The new virus is already part of the biological diversity that human influenza viruses represent. As this new strain will continue to coexist with us, we must pay attention to its evolution and, if necessary, make the decisions leading to assure the health of the population.

## Lessons learned

There is a series of lessons we can draw from the influenza epidemic.

1. The possibility of effective and timely action in the presence of the sanitary contingency was only possible thanks to the decided support of the President of the Republic. With the available evidence, he instructed and empowered the Ministry of Health to act singly at the national level in accordance with the current legal framework. The action involved the entire force of the Mexican State.

2. The early planning that resulted from the National Pandemic Influenza Preparedness and Response Plan set the foundations for timely action. However, the operational problems that arose could not have been solved without the support of other Secretaries of State pertaining to the labor, education, social development, agriculture, economics, and treasury sectors, all of whom acted in a solidary manner. The epidemic led to identifying operational implications that are being addressed and that will, in turn, permit our preparedness in case of a pandemic or a new epidemic outbreak.

3. The coordination among all the government levels was achieved mainly through the States' Secretaries of Health by means of extraordinary meetings of the National Health Council and the General Health Council, and led to a joint decision making process of the government and the society to abate this epidemic. Despite the great coordination achieved, there were information gaps concerning the course of the epidemic and particularly the data updating process, but this situation was addressed with measures systematizing the information routes and cutoff dates. We are implementing provisions to strengthen the timeliness and reliability of the epidemiologic information.

4. The implementation of preventive measures that precluded a major contingency from occurring was possible thanks to the maturity with which all Mexicans behaved and their trust in governmental decisions. The trust of the population resulted in part from the fact that Mexicans were permanently informed with transparency and formality throughout the different stages of the epidemic, in real time. Moreover, the international organizations involved certified the data generated on a daily basis and endorsed the actions implemented by the federal government.

5. The presence and solidarity of international organizations, foreign governments and private businesses provided the technical support that facilitated both confirming the presence of a new virus and the access to the scientific knowledge accrued internationally. Additionally, the in-kind contributions increased the country's stores of medications and health supplies, thus helping to lessen the contingency.

6. The rest of the world benefited from the Mexican experience thanks to the timeliness with which the government issued the sanitary alert and informed about the course of the epidemic. The knowledge shared by Mexico provided the countries who have faced the human influenza virus A(H1N1) with information about its epidemiologic characteristics, its clinical picture and the chance of cure if timely healthcare is provided. This, in turn, has led to mitigating the economic, sanitary and social effects. Upon donating the strain of this new virus to the world through WHO, Mexico has contributed to developing a specific vaccine that will make it possible to prevent and control this new variety of human influenza. Moreover, the country has given proof of solidarity and responsibility for global health even at a time of a major economic crisis.

7. Even though quick and effective action was taken, the need to strengthen the epidemiologic surveillance systems, the laboratories and the information networks became clear to us. We also need to update the legislation so that it allows us to act appropriately under pandemic situations, facilitate vaccine production and distribution, and promote the national scientific research on priority health topics.

8. As mental health is not a minor issue, mental health programs specific for sanitary emergencies are necessary to address their psychological effects on the healthcare personnel, patients, their families and the general population. The latter effects, however, did not occur during this pandemic because the people were oriented and referred accordingly through the toll-free number.

9. The epidemic had a positive effect on the population from the perspective of the personal and environmental preventive hygiene measures. We therefore constantly urge the citizenship to turn the latter into a healthy habit that becomes part of self-care and to adopt other healthy personal habits.

The emergence of the new influenza virus will undoubtedly become a public health landmark and will be recorded in the annals of world history. Those of us who have the opportunity of living this unique experience today must learn from it to strengthen the capabilities and overcome the weaknesses of our healthcare systems. In doing so, this new resident of the global world will cause the least possible havoc.

The influenza A(H1N1) epidemic in Mexico has not finished yet, and currently we continue been in permanent alert to face the next winter season period.
